# Lower body kinematic changes induced by anterior cruciate ligament transection: an *in vivo* three-dimensional analysis in rats

**DOI:** 10.7717/peerj.21016

**Published:** 2026-03-23

**Authors:** Momoko Nagai-Tanima, Hideki Kawai, Aoi Ishikawa, Kanon Ishida, Hiroshi Kuroki, Tomoki Aoyama

**Affiliations:** Department of Human Health Sciences, Graduate School of Medicine, Kyoto University, Kyoto, Japan

**Keywords:** Anterior cruciate ligament, Rat, *In vivo*, Kinematics, Animal model

## Abstract

**Background:**

Gait disturbance is readily recognized as a disruption in walking performance, and gait analysis offers insight into the functional impairment of all lower leg joints. However, limited information is available on three-dimensional (3D) kinematic analysis of the lower leg following anterior cruciate ligament transection (ACLT) in rodent models. This information obtained using animal models contributes to the establishment of effective interventions with a view to clinical application. This study aimed to clarify the 3D kinematic alterations and compensatory actions of joints adjacent to the knee, as well as their correlations, in a rat model.

**Methods:**

Forty male Wistar rats were assigned to either the control (Ctrl) or ACLT group. Three-dimensional motion analysis was conducted at 1, 2, 4, and 8 weeks post-surgery. Two types of assessment parameters were analyzed based on the plane of assessment: sagittal (2D) and other (3D). Lower limb joint angles during gait were measured, and the relationship between knee joint changes and alterations in other joints was analyzed.

**Results:**

An interaction between group and time was observed not only in knee range of motion (ROM), but also in adjacent hip ROM. Hip ROM was significantly reduced in the ACLT group compared with the Ctrl group at 8 weeks (*p* < 0.05). Additionally, a correlation between hip ROM and knee parameters was noted at 8 weeks. Among them, hip flexion range was positively correlated with knee valgus and knee flexion range (*r* = 0.87, *p* = 0.03; *r* = 0.84, *p* = 0.03, respectively). No interaction was observed in the 3D parameters.

**Conclusion:**

The findings of this study demonstrate a compensatory response in the hip joint of rats after ACLT, which parallels observations in individuals with anterior cruciate ligament injuries. This may offer further insight into the impact of ACL injuries on adjacent joints and inform rehabilitation strategies.

## Introduction

Anterior cruciate ligament (ACL) injury induces knee joint instability (Alkjaer et al., 2003; Andriacchi & Dyrby, 2005) and increases the risk of post-traumatic conditions such as knee osteoarthritis (KOA) by altering the location and magnitude of load-bearing regions within the articular cartilage (Andriacchi & Dyrby, 2005; Crook et al., 2021) through kinematic changes (Daniel et al., 1994; Lohmander et al., 2004). Altered knee joint motion occurs immediately following ACL injury (Hurd & Snyder-Mackler, 2007), also affecting the movement patterns of adjacent joints (Torry et al., 2004; Hurd & Snyder-Mackler, 2007; Chaudhari, Schmitt & Andriacchi, 2012; Wellsandt et al., 2017). Abnormal movement in adjacent joints may contribute to the increased risk of joint cartilage degeneration.

Biomechanical changes in the proximal and distal joints within the kinematic chain presumably act to compensate for altered knee joint motion after ACL injury (Hurd & Snyder-Mackler, 2007). ACL-deficient knees exhibit anterior tibial translation in both humans (Daniel et al., 1994; Georgoulis et al., 2003) and other species (Tinga et al., 2020; Bascuñán et al., 2021). Several studies have reported altered hip joint movement patterns in the injured limb after ACL injury in humans (Ferber et al., 2002; Di Stasi et al., 2013; Wellsandt et al., 2017). Ferber et al. (2002) hypothesized that changes in hip motion may serve as a compensatory mechanism to reduce anterior tibial translation. The same phenomenon may occur during conservative treatment following an ACL rupture, potentially resulting in secondary injuries. However, there are few reports investigating treatment approaches for the secondary damage on other joints caused by biomechanical alteration following conservative management after ACL injury. Although, early detection and intervention are still considered desirable for preventing secondary disorders such as post-traumatic osteoarthritis (OA) (Chang et al., 2017; Wang et al., 2020), the prognosis of conservative treatment is affected by various factors (Wang et al., 2020) and variations between the onset of the symptoms and the disease in humans (Karsdal et al., 2014), making it challenging to establish standardized treatments in humans. If compensatory movements observed in humans are also demonstrated in animal models, animal models may be applicable for investigating and developing therapeutic approaches. Therefore, understanding the kinematics of compensatory movements using an animal model is a crucial first step toward developing preventive therapeutic interventions for human disorders caused by such movements.

Animal models of anterior cruciate ligament transection (ACLT) have been used to investigate the molecular and morphogenetic mechanisms underlying osteoarthritis (Fu et al., 2012; Ruan et al., 2013). Also, kinematic analysis during gait using the model has also been conducted to understand the pathology of KOA as influenced by mechanical stress alterations on cartilage. Regarding OA research using animal models, various biomechanical parameters in gait analysis (such as spatiotemporal, kinematic, and mechanical factors) are interrelated and contribute to compensatory movements (Lakes & Allen, 2016; Chan, Bowe & Allen, 2023). Three-dimensional (3D) motion capture enables the visualization of joint movement in 3D space (Tajino et al., 2015; Iijima et al., 2016) serves as a powerful tool for advancing our understanding of gait kinematics (Lakes & Allen, 2016). The animals used for the kinematics research after ACLT vary, including rodents (Ferland et al., 2011; Ängeby Möller et al., 2020), dogs (Tinga et al., 2020), goats (Bascuñán et al., 2021) and sheeps (Barton et al., 2019) and more. Most prior studies have focused on the relationship between morpho-pathological alterations of the cartilage and knee joint kinematic alterations including joint force, or knee joint parameters including the angle and the moment in various directions. However, no study has yet characterized joint movement patterns in the lower limb after ACL injury in an animal model from a compensatory perspective.

The present study aimed to clarify the 3D kinematic changes, the compensatory actions of joints adjacent to the knee, and the correlation between these changes in an ACLT rat model.

## Materials and Methods

### Animals

**Figure 1:**
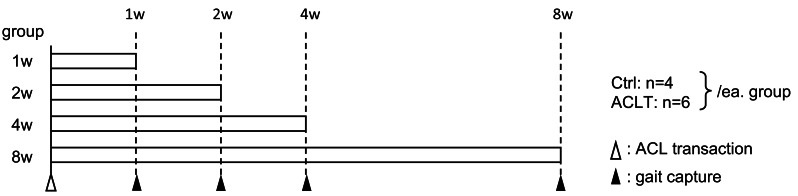
Experimental schema. At each time point, gait capture was conducted on four Ctrl and six ACLT rats. Ctrl, control; ACLT, anterior cruciate ligament transection.

Forty 12-week-old male Wistar rats (Japan SLC, Tokyo, Japan) were randomly and evenly divided into groups for analysis at four specific time points: 1, 2, 4, and 8 weeks. The sample size for an effect size of 0.6, significance level of 0.05, power of 0.8, and five-group comparison was 39, so the sample size was determined based on this calculation. The effect size was determined based on pre-experimental data on knee joint ROM, which is significantly affected by the intervention in this experiment. In addition, the sample size was determined with reference to similar studies (Barton et al., 2019; Suter et al., 1998). To demonstrate changes over time, the rats were analyzed during an acute to semi-long experimental period. Subsequently, the animals were randomly divided into two groups: the ACLT and control (Ctrl) groups, with six and four rats at each time point respectively, using a simple randomization method. Randomization was performed manually by drawing lots prepared before the start of the experiment by an investigator. Analyses were performed in a blinded manner by the same experimenter who performed the randomization. The Ctrl group was used as an independent intact group, since the contralateral limb may be kinematically influenced by the intervention on the affected limb (Iijima et al., 2016). The experimental design is shown in Fig. 1. All animals were housed in pairs (two animals per cage, cage size: 260  ×  420  × 150 mm) at room temperature (23 °C) with a 12 h:12 h light: dark cycle, and food and water were provided *ad libitum*. All procedures were approved by and conducted in accordance with the guidelines of the Kyoto University Animal Experimentation Committee (Approval number: Med Kyo 21581). The exclusion criteria included animals that developed surgical site infections or worsening inflammation before the end of the experiment; however, no animals met these criteria during the study period. After all analysis for this experiment, rats were anesthetized with isoflurane and sacrificed by exsanguination and excised tissues were used for analyses outside of this study.

### Surgical procedures and animal care

In the ACLT group, surgical operations were performed on the right hind limb in accordance with previous reports (Stoop et al., 2001). In brief, the animals were anesthetized *via* intraperitoneal injection of a mixed anesthetic comprising 0.15 mg/kg medetomidine, 2 mg/kg midazolam, and 2.5 mg/kg butorphanol. After shaving the knee joint, the skin was disinfected with iodine. A longitudinal incision was made on the medial side of the right knee, and the medial capsule was exposed by laterally dislocating the patella. The ACL was completely transected with small surgical scissors at the medial intercondylar eminence of the tibia. Knee joint instability was subsequently confirmed manually using anterior traction of the tibia (Murata et al., 2017).

### Three-dimensional gait capture apparatus and data acquisition

**Figure 2:**
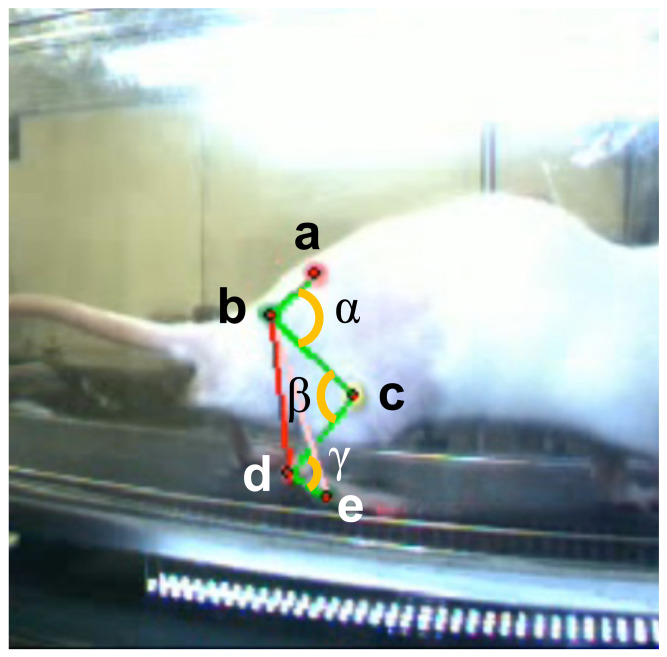
Landmarks of motion capture and joint angles on sagittal plane. Notes: Anterior superior iliac spine (a), trochanter major, b; knee joint, c; lateral malleolus, d; 5th metatarsophalangeal joint, e; hip flexion angle, *α*; knee flexion angle, *β*; knee flexion angle, *γ*.

Gait capture was conducted at each time point for both ACLT and Ctrl groups. The kinematic properties of both hind limbs during treadmill ambulation were recorded using a 3D motion capture system (Kinema Tracer; Kissei Comtec, Nagano, Japan). The treadmill speed was set at 8.5 m/min for each session, modified from the standard speed (12 m/min) (Tajino et al., 2015) because ACLT rats could not maintain running at that speed at 1 week post-surgery. The selected speed was verified by confirming that all rats could sustain ambulation under these conditions. Details of the apparatus and measurement configuration for knee kinematics during gait are described in our previous reports (Tajino et al., 2015). In brief, using 3D motion capture analysis of rat locomotion, we defined five landmarks on each hind limb: anterior superior iliac spine (ASIS); trochanter major (hip); knee joint (knee); lateral malleolus (ankle); and 5th metatarsophalangeal joint (MTP) (Fig. 2). Under isoflurane anesthesia, landmarks markers were attached to the body and removed after measurement. Five consecutive steps of the right hind limb were used in the data set for each rat. The trajectory of each angle was automatically extracted, defined as the angle formed by connecting lines, as described in the following section.

Based on gait analysis data, the chronological alteration in lower limb joint angles (hip, knee, and ankle joints) during gait were analyzed to capture the effects of the intervention on each joint. Using these results, compensatory movements were examined through the following two-step approach: (1) To identify the factors influencing each joint range of motion (ROM) change, a two-way ANOVA was performed to assess the effects of intervention and time. *Post-hoc* tests were conducted for factors where significant differences were observed; when an interaction was observed, it was assumed that other factors had an influence and moved on to the next analysis step. (2) For parameters showing interactions, correlations with knee-related factors were analyzed. Details are provided below.

### Motion parameters in locomotion

We evaluated several parameters to characterize the gait motion of the ACLT model, which were categorized into two groups: sagittal (2D) and 3D-specific parameters (Fig. S1). The sagittal (2D) group included metrics that could be observed within the sagittal plane. The 3D-specific group referred to parameters in the coronal or horizontal planes that were unique to the 3D reconstructed motion components. The ROM was calculated by subtracting the minimum angle from the maximum angle throughout both the stance and swing phases.

#### 2D parameters

2D parameters were derived from the hip, knee, and ankle angles during gait cycles. The hip angle was defined by a line connecting the “ASIS” to the “hip” and a line connecting the “knee” to the “hip”. The knee angle was defined by a line connecting the “hip” to the “knee” and a line connecting the “ankle” to the “knee”. The ankle angle was defined by a line connecting the “knee” to the “ankle” and a line connecting the “MTP” to the “ankle”. For hip angles, a greater angle indicated plantarflexion (Fig. 2). For knee angles, a greater angle indicated extension. For ankle angles, a greater angle indicated reduced dorsiflexion. To investigate the load response of the ankle joint, the ankle angle was analyzed at midstance of the right leg, defined as the moment when the relevant limb was passed by the opposite swinging leg.

#### 3D specific parameters

3D parameters were derived from the reconstructed properties of the processed data. To capture the compensatory kinematics induced by ACLT, we evaluated them as follows: The pelvic tilt angle was defined as the angle between the horizontal line and the line connecting the right and left ASIS markers on the coronal plane. Positive and negative values represented a tilt toward the left and right sides, respectively. The hip abduction/adduction angle was formed by a line connecting the “hip” and “knee” and a reference line on the coronal plane, defined as the perpendicular line between the hip joint and the floor. The knee inversion/valgus angle was formed using the same lines as the knee angle, measured on the horizontal plane.

### Statistical analysis

Normality of each data distribution was confirmed by Shapiro–Wilk test. For the purpose of comparison with previous studies, maximum and minimum flexion angle parameters of the hip, knee, and ankle joints were compared between ACLT and control using unpaired t-tests. A two-way ANOVA was conducted to examine the effects of “Group” and “Week” on each ROM parameter. Parameters with interactions were tested for simple effects and were hypothesized to involve factors beyond the main effect. For parameters with significant interactions, we assessed correlation strength with other variables using Pearson correlation coefficients. We used unpaired t-tests to determine differences in the parameters between the Ctrl and ACLT groups. All analyses were performed using JMP Pro Version 16 (SAS Institute Inc., Cary, NC, USA). 3D parameters are presented as the mean and standard deviation. Statistical significance was set at *p* < 0.05.

## Results

### Results of two-way ANOVA for each parameter

The two-way ANOVA revealed significant main effects of the Group factor on Knee flexion ROM parameter in 2D parameters (F(1,32) = 18.72, *p* = 0.001). Additionally, a significant interaction effect between Group and Week was found for the Knee flexion and Hip flexion ROM parameters (Knee: F(3,32) = 5.12, *p* = 0.005; Hip: F(3,32) = 5.68, *p* = 0.003). For the 3D parameter, only a significant main effect of the Group was found for the Knee inversion/valgus parameter (F(1,32) = 13.25, *p* = 0.001). There was no main effect of the Week factor for any of the 2D or 3D parameters (Table 1).

**Table 1:** Results of each ROM parameters on two-way ANOVA.

		2D parameters					
Angle		Hip flex ROM		Knee flex ROM		Ankle flex ROM	
		F-value	*p*-value	F-value	*p*-value	F-value	*p*-value
Group		2.012	0.166	18.721	*p* < 0.01^*^	2.608	0.116
Week		2.262	0.100	1.972	0.138	2.070	0.124
Group*week		5.682	*p* < 0.01^*^	5.117	0.005^*^	1.731	0.181

		3D parameters					
Angle		Pelvic tilt		Hip Abd/Add		Knee Inv/Val	
		F-value	*p*-value	F-value	*p*-value	F-value	*p*-value
Group		0.970	0.332	0.927	0.343	13.247	0.001^*^
Week		1.171	0.336	0.188	0.904	1.599	0.209
Group*week		0.616	0.610	1.290	0.295	1.558	0.219

**Notes.**

* *P* < 0.05.

### 2D parameter characteristics in time comparison

In summary, the knee, ankle, and hip 2D parameters showed a trend toward recovery during the experimental period. However, the hip showed deterioration after recovery at 8 weeks post-operation.

**Figure 3:**
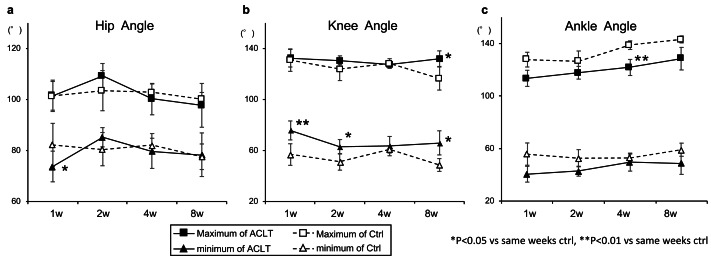
Maximum and minimum angle of the hip and knee joints. (A) Hip; (B) knee and (C) ankle angles. The maximum (squares) and the minimum (triangles) angles during gait for the control (open symbols) and the experimental (closed symbols) hind limbs at different time points. Significant differences between the control and ACLT groups at each time point are indicated by asterisks (*p < 0.05, **p < 0.01). Data are shown as mean values ± SD. ACLT, anterior cruciate ligament transection; SD, standard deviation.

**Figure 4:**
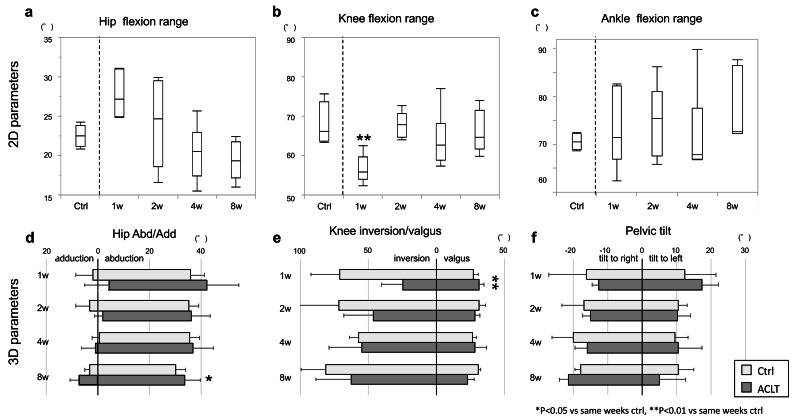
2D and 3D gait parameters obtained from the kinema-tracer. 2D parameters, including (A) hip flexion range; (B) knee flexion range; and (C) ankle flexion range were analyzed. As a representative, the Ctrl at eight weeks is shown in each graph. The six 3D parameters were divided into three related joints: (D) hip adduction/abduction; (E) knee inversion/valgus; and (F) pelvic tilt to right/left. Significant differences between the Ctrl and ACLT groups at each time point for ROM results are indicated by asterisks (*p < 0.05, **p < 0.01). Data are shown as the median and interquartile range (IQR) on A, B and C, and mean values ± SD on D, E and F. Ctrl, control; ACLT, anterior cruciate ligament transection; SD, standard deviation.

Regarding the hip joint, the maximum and minimum angles were lower in the ACLT group than in the Ctrl group, except at 2 weeks; however, this difference was only significant for the minimum angle at 1 week (Ctrl, 82.17 ± 3.70 *vs.* ACLT, 73.73 ± 6.03; *p* < 0.05; Fig. 3A). The range of the hip joint in the ACLT group increased in the early phase, decreasing gradually thereafter (1 week: Ctrl, 19.36 [IQR, 16.51–21.90] *vs.* ACLT, 27.16 [IQR, 24.95–31.02]; 2 weeks: Ctrl, 23.18 [IQR, 20.42–25.23] *vs.* ACLT, 24.70 [IQR, 18.62–29.47]; 4 weeks: Ctrl, 20.84 [IQR, 19.73–22.12] *vs.* ACLT, 20.49 [IQR, 17.43–22.92]; 8 weeks: Ctrl, 22.53 [IQR, 21.17–23.86] *vs.* ACLT, 19.34 [IQR, 17.19–21.77]; Fig. 4A, Fig. S2A). Regarding the knee joint, the ACLT group showed significantly higher minimum angles than the Ctrl throughout the experimental period, except at 4 weeks (1 week: Ctrl, 56.83 ± 8.47 *vs.* ACLT, 75.72 ± 7.43; *p* < 0.01; 2 weeks: Ctrl, 51.10 ± 6.52 *vs.* ACLT, 62.92 ± 5.65; *p* < 0.05; 8 weeks: Ctrl, 48.62 ± 5.01 *vs.* ACLT, 66.00 ± 9.46; *p* < 0.05), and significantly higher maximum angle at 8 weeks (Ctrl, 116.47 ± 9.05 *vs.* ACLT, 132.0 ± 6.10; *p* < 0.05; Fig. 3B). The ROM in the ACLT group was significantly decreased compared to the Ctrl at 1 week (Ctrl, 73.60 [IQR, 68.12–80.48] *vs.* ACLT, 55.89 [IQR, 54.00–59.60]; *p* < 0.01, Fig. 4B). Thereafter, an increasing trend was observed at 2 weeks and was maintained until 8 weeks (2 weeks: Ctrl, 72.22 [IQR, 69.84–75.83] *vs.* ACLT, 67.80 [IQR, 64.59–70.72]; 4 weeks: Ctrl, 67.95 [IQR, 65.82–68.56] *vs.* ACLT, 62.68 [IQR, 58.86–68.15]; 8 weeks: Ctrl, 66.22 [IQR, 63.61–73.74] *vs.* ACLT, 64.71 [IQR, 61.71–71.56]). After 2 weeks, there was no significant difference in the range between ACLT and Ctrl at each time point (Fig. 4B). The representative trajectory of the knee joint showed a higher angle in the ACLT group than in the Ctrl group throughout the gait cycle at 1 week (Fig. S2B). Regarding the ankle joint, the ACLT group showed lower maximum and minimum angles than the Ctrl throughout the experimental period and a significantly lower maximum angle at 4 weeks (Ctrl, 138.79 ± 6.70 *vs.* ACLT, 121.78 ± 6.72; *p* < 0.05; Fig. 3C). There was no significant alteration in the range of the ankle joint during the experimental period (1 week: Ctrl, 70.52 [IQR, 63.63–81.85] *vs.* ACLT, 71.40 [IQR, 66.95–82.19]; 2 weeks: Ctrl, 73.87[IQR, 71.26–76.42] *vs.* ACLT, 75.39 [IQR, 67.58–80.98]; 4 weeks: Ctrl, 89.55 [IQR, 74.19–93.55] *vs.* ACLT, 67.80 [IQR, 66.97–77.57]; 8 weeks: Ctrl, 81.35 [IQR, 77.41–93.39] *vs.* ACLT, 72.64 [IQR, 72.21–86.39]; Fig. 4C). The raw data was shown in Tables S1, S2.

### 3D parameter characteristics in time comparison

In summary, the ACLT group showed a wider hip abduction/adduction range than the Ctrl at 8 weeks, with progressively increasing trend knee inversion/valgus ranges from 1 week to 8 weeks. Although there was no statistically significant difference in pelvic tilt, a tendency toward a rightward shift was observed.

Regarding the hip joint, the abduction angle in the ACLT group trended higher than in the Ctrl during the experimental period. There were no significant differences between time points. The trend in the adduction angle in the ACLT group was lower than that of the Ctrl in the early phase; however, this trend inverted after 4 weeks. The ROM in the ACLT group was significantly higher than that in the Ctrl at 8 weeks (Ctrl, 33.34 ± 2.07 *vs.* ACLT, 40.97 ± 4.75; *p* < 0.05; Fig. 4D). The representative trajectory of hip abduction/adduction showed that the peak abduction angle (maximum) during the swing phase and the peak adduction angle (minimum) during the stance phase were both higher in the ACLT group than in the Ctrl at 8 weeks (Fig. S2C). Regarding the knee joint, the valgus angle in the ACLT group was not significantly changed during experimental period. The inversion angle in the ACLT group was lower than that of the Ctrl early on and increased gradually over time. Although the total knee inversion/valgus range in the ACLT group was significantly smaller than in the Ctrl group at 1 week (Ctrl, 97.98 ± 24.49 *vs.* ACLT, 56.21 ± 14.55; *p* < 0.01), it gradually increased throughout the experimental period (Fig. 4E). The representative trajectory of knee inversion/valgus in the ACLT group showed a lower peak and smaller range than in the Ctrl at 8 weeks (Fig. S2D). Regarding pelvic tilt, the leftward tilt in the ACLT group gradually decreased over the experimental period. The rightward tilt in the ACLT group increased gradually. However, there was no significant difference between the Ctrl and ACLT groups at any time point, or between time points (Fig. 4F). No significant differences between time points were found for any 3D parameter in the Ctrl group. The raw data was shown in Table S3.

### Correlation between parameters

Significant correlations between Hip flexion ROM or knee parameters and other parameters are shown in Table 2. In summary, the knee flexion range was related to hip or knee 3D parameters throughout the experimental period, except at 4 weeks. The hip flexion range also showed correlations with several other parameters during the entire experimental period, except at 4 weeks.

**Table 2:** Correlations between parameters at each time point.

**1w**	Hip adduction								
	*r*	*p*-value							
Hip flexion range	0.86	0.026^*^							
Knee flexion range	−0.85	0.030^*^							

**2w**		Hip adduction		Pelvic tilt to Left		Pelvic tilt to Right		Knee inversion		
		*r*	*p*-value	*r*	*p*-value	*r*	*p*-value	*r*	*p*-value	
Hip flexion range		0.85	0.032^*^	0.82	0.047^*^	0.94	0.004^*^	0.03	0.96	
Knee flexion range		−0.58	0.23	−0.81	0.05	−0.45	0.37	−0.87	0.024^*^	

**4w**	Ankle flexion range								
	*r*	*p*-value							
Knee valgus	0.87	0.024^*^							

**8w**		Knee valgus		Knee inversion		Hip flexion range		Ankle flexion range		
		*r*	*p*-value	*r*	*p*-value	*r*	*p*-value	*r*	*p*-value	
Hip flexion range		0.87	0.025^*^	−0.89	0.018^*^			−0.94	0.019^*^	
Knee flexion range		0.70	0.12	−0.88	0.020^*^	0.84	0.035^*^	−0.76	0.14	

**Notes.**

* *P* < 0.05.

**Figure 5:**
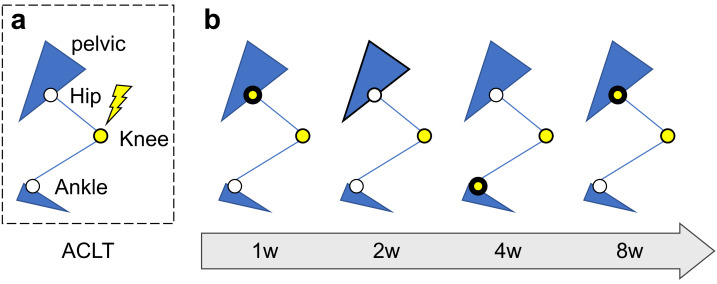
Schematic diagram of time-course changes in compensatory joints related with knee joint. (A) Schematic image of the sagittal plane of the rat lower limbs with anterior cruciate ligament transection; (B) Time series changes in compensatory joints after ACLT. Joints with significant correlations with knee joint parameters are indicated by yellow circles surrounded by thick lines. The knee joint is indicated by yellow circles.

At 1 week, there was a negative correlation between the knee flexion range and hip adduction (*r* = −0.85, *p* = 0.03). Additionally, there was a positive correlation between the hip flexion range and hip adduction (*r* = 0.86, *p* = 0.03). However, no correlation was observed between the hip flexion range and knee 3D parameters. At 2 weeks, the knee flexion range was negatively correlated with knee inversion (*r* = −0.87, *p* = 0.02). The hip flexion range was positively correlated with hip adduction and pelvic tilt in both directions (hip adduction: *r* = 0.85, *p* = 0.03; pelvic tilt to left: *r* = 0.82, *p* = 0.05; to right: *r* = 0.94, *p* = 0.004). At 4 weeks, a positive correlation was observed between knee valgus and ankle flexion range (*r* = 0.87, *p* = 0.03). At 8 weeks, hip flexion range was positively correlated with knee valgus (*r* = 0.87, *p* = 0.03). Conversely, hip flexion range was negatively correlated with knee inversion and ankle flexion range (*r* = −0.89, *p* = 0.018; *r* = −0.94, *p* = 0.0019, respectively). A negative correlation was also observed between knee flexion range and inversion (*r* = −0.88, *p* = 0.02), while a positive correlation was found between hip and knee flexion range (*r* = 0.84, *p* = 0.03). Schematic diagram of time-course changes in compensatory movements is shown in Fig. 5.

## Discussion

The present study was conducted to clarify the 3D kinematic changes and compensatory actions of adjacent joints, as well as the correlations between these changes, following ACLT in a rat model. To demonstrate alterations over time, model rats were analyzed from the acute to semi-long experimental period. Considering the kinematic chain, we hypothesized that hip joint or adjacent joint movement alterations would be induced by kinematic changes following ACLT of the knee joint. Interestingly, this study revealed an interaction between group and intervention duration not only in knee ROM but also in adjacent hip ROM. Furthermore, a correlation between hip ROM and knee parameters was also observed at 8 weeks postoperatively. This indicates that kinematic alterations were induced not only in the knee joint but also in the adjacent joints at 8 weeks by ACLT intervention in a rat model.

Several reports have previously discussed gait characteristics following ACLT in animal models. Bascuñán et al. (2021) reported that knee extension developed in goats at 3 months post-operation compared with baseline. A cat ACLT model reported that, although hip flexion angle decreased after ACLT, no significant decrease was observed. Furthermore, the ankle maximum flexion angle in the Ctrl group was significantly higher than that of the ACLT group at 3 weeks post-operation, with no significant difference in the minimum angle during this period (Suter et al., 1998). The current study showed the same trend as these studies. Despite differences in animal species, the trends in lower limb joint angle changes were similar among quadrupedal animals.

Regarding the knee joint, quadriceps avoidance is described as a kinematic adaptation resulting from anterior tibial translation to minimize quadriceps contraction (Wexler et al., 1998). The decreased knee flexion during gait has been observed in humans with ACL deficiency (Torry et al., 2004). Furthermore, quadriceps—hamstring muscle co-contraction has also been reported as a positive adaptation after ACLT injury (Alkjaer et al., 2003; Hurd & Snyder-Mackler, 2007). This strategy helps stabilize the knee in the absence of ligamentous support. These patterns may support the knee extension observed in the current study. The lower leg skeleton and the ROM of the knee joint differ between species. Additionally, the rat knee joint typically remains flexed while standing and walking, in contrast to humans and other large animals (Oláh et al., 2021). Despite these skeletal differences, knee extension was also confirmed in our rat ACLT model.

In terms of hip joint parameters, an interaction between group and intervention duration was observed in hip ROM. A correlation between hip parameters and knee parameters was also identified at 1 and 8 weeks postoperatively, with a notable correlation between hip ROM and knee parameters at 8 weeks. In humans, increased hip extensor output has been demonstrated in ACL-deficient individuals (Stoop et al., 2001); this trend was also confirmed in the present study. The movement patterns and biomechanical changes in proximal and distal joints within the leg kinetic chain may arise due to compensation for knee joint kinematic alterations after ACL injury (Hurd & Snyder-Mackler, 2007). The hip provides the largest contribution to the total support moment during weight acceptance (Hurd & Snyder-Mackler, 2007). In practice, alterations in the movement of the hip joint after ACL rupture have been described in several human studies (Torry et al., 2004; Hurd & Snyder-Mackler, 2007; Chaudhari, Schmitt & Andriacchi, 2012; Di Stasi et al., 2013; Wellsandt et al., 2017). ACL-deficient individuals show decreased hip flexion range and increased hip adduction range during gait compared to controls, even 20 years post-injury (Markström, Tengman & Häger, 2018). Analyses of the support moment contribution after acute ACL rupture show increased hip weight acceptance (Hurd & Snyder-Mackler, 2007). These findings indicate that the hip joint compensates for the knee kinematic alterations induced by ACL rupture. A similar reaction of the hip joint was observed in the current study. Therefore, this compensatory reaction of the hip joint is not surprising in humans, but it is of great significance that this finding could be reproduced in a rat model as well.

In terms of ankle joint parameters, no significant results were found in the two-way ANOVA analysis in the ankle ROM; however, correlations were found between knee valgus parameters at 4 weeks and hip joint ROM at 8 weeks. In previous biomechanical studies involving individuals with ACL deficiency, no significant effects were observed on ankle joint angles or torques during gait (Torry et al., 2004; Lindström et al., 2010), and a compensatory strategy involving increased reliance on the hip joint has been reported (Torry et al., 2004). Consistent with these findings, the present study did not reveal any correlation between the ankle joint and specific joints, nor was there a correlation with knee joint parameters in the early phase of intervention. Therefore, the correlations observed are considered to be transient.

Regarding the 3D parameters, a significant difference in the two-way ANOVA result was observed only in knee Inv/Val between groups, while no significant main effects of group or time, nor any interaction effects, were found for the other 3D parameters. This indicates that the intervention had no observable effect on the coronal or frontal plane movements of the hip or pelvis. The finding that knee inversion/valgus increases following ACL rupture is consistent with previous studies in other animal models (Barton et al., 2019; Bascuñán et al., 2021). Although there are no existing reports concerning pelvic or hip abduction/adduction in other animals, considering the function of the ACL, the restraint of anterior tibial translation, it is plausible that the impact of ACL deficiency predominantly affects sagittal plane dynamics in adjacent joints.

Our study has some potential limitations. First, the controls in this study did not undergo a sham operation to obtain the kinematic features of an intact condition control. Therefore, it is possible that the gait in the early postoperative period is influenced not only by the intervention but also by pain resulting from the surgery. Second, individual subjects were used for each time point. Longitudinal analysis using the same animal for comparison between baseline and the results of each time point could reveal the chronological effect in the same subject and may avoid this dispersion of results. Third, although the analysis was blinded, it was the same experimenter who randomized the samples, which may have introduced bias into the analysis. Forth, we primarily focused on the joint angle in the current study. In the future, analyses of moment data and other biomechanical parameters are expected to provide valuable insights that will further enhance our understanding of kinematic changes in animal model experiments.

## Conclusion

The findings of this study demonstrated that a compensatory response is observed after ACLT in a rat animal model, and that the compensatory joint changes over time. In particular, the compensatory movements at the hip joint, was consistent with observations in humans with anterior cruciate ligament injury. This may offer further insight into the impact of ACL injuries on adjacent joints and inform rehabilitation strategies.

##  Supplemental Information

10.7717/peerj.21016/supp-1 Supplemental Information 1 Raw data

10.7717/peerj.21016/supp-2 Supplemental Information 2 Raw data of Figure 3 Data unit: degree.

10.7717/peerj.21016/supp-3 Supplemental Information 3 Raw data of joint flexion range for 2D parameters in Figure 4

10.7717/peerj.21016/supp-4 Supplemental Information 4 Raw data of 3D parameters in Figure 4 Negative values represent numerical values in three-dimensional space and were switched to positive values for analysis. *p < 0.05 and **p < 0.01 indicate statistical significance compared with the control group in the same week for the range results. All data unit is degree.

10.7717/peerj.21016/supp-5 Supplemental Information 5 Parameters of gait analysis (a) 2D parameters on the sagittal plane; and (b) 3D parameters on the coronal and horizontal planes. The yellow circular line indicates the measured angle. On the sagittal plane, three parameters: hip flexion, knee flexion, and ankle angle, were analyzed. On the coronal plane, two parameters: pelvic tilt to the right/left and hip abduction/adduction were analyzed. On the horizontal plane, knee inversion/valgus were analyzed.

10.7717/peerj.21016/supp-6 Supplemental Information 6 Representative trajectory of joint parameters on the lower leg (a) The representative trajectory of hip flexion range at eight weeks showing a similar trajectory and lower angle range in the ACLT group compared to the Ctrl. (b) The representative trajectory of knee flexion range at one week showing a higher angle and lower angle range in the ACLT group compared to the Ctrl. (c) The trajectory of hip abduction/adduction at eight weeks showing the peak of the abduction angle (maximum) in the swing phase and peak of adduction angle (minimum) in the stance phase of ACLT are larger than that of the Ctrl. (d) The trajectory of knee valgus/inversion range at eight weeks showing lower inversion and valgus angles and a lower angle range in the ACLT group compared to the Ctrl.

10.7717/peerj.21016/supp-7 Supplemental Information 7 ARRIVE 2.0 checklist

## Additional Information and Declarations

### Data Availability

The following information was supplied regarding data availability: The raw data is available in the Supplemental Files.
