# Supplementary material for: Lower body kinematic changes induced by anterior cruciate ligament transection: an in vivo three-dimensional analysis in rats
Source: PeerJ. 2026 Mar 23;14:e21016. doi: 10.7717/peerj.21016 (PMC13020435; doi:10.7717/peerj.21016)
Supplement: Supplemental Information 2 — Data unit: degree. [file peerj-14-21016-s002.docx]

Supplemental Table 1. Raw data of Figure3.

|  | **Hip flex** | | | | **Knee flex** | | | | **Ankle flex** | | | |
| --- | --- | --- | --- | --- | --- | --- | --- | --- | --- | --- | --- | --- |
|  | Ctrl | | ACLT | | Ctrl | | ACLT | | Ctrl | | ACLT | |
| **Max** | mean | SD | mean | SD | mean | SD | mean | SD | mean | SD | mean | SD |
| 1w | 101.42 | 5.61 | 101.44 | 6.14 | 130.89 | 8.78 | 132.36 | 6.85 | 127.78 | 16.62 | 113.53 | 11.67 |
| 2w | 103.46 | 7.87 | 109.25 | 4.85 | 123.73 | 8.87 | 130.78 | 3.43 | 126.51 | 9.95 | 117.78 | 20.31 |
| 4w | 102.81 | 3.30 | 100.16 | 6.15 | 128.30 | 3.69 | 127.48 | 3.05 | 138.79 | 6.70 | 121.78** | 6.72 |
| 8w | 100.11 | 2.65 | 97.71 | 8.59 | 116.47 | 9.05 | 132.0* | 6.10 | 143.20 | 5.36 | 128.53 | 13.89 |
| **min** |  |  |  |  |  |  |  |  |  |  |  |  |
| 1w | 82.17 | 3.70 | 73.73* | 6.03 | 56.83 | 8.47 | 75.72** | 7.43 | 55.78 | 7.53 | 40.47 | 12.39 |
| 2w | 80.52 | 6.78 | 85.19 | 3.78 | 51.10 | 6.52 | 62.92* | 5.65 | 52.66 | 8.60 | 42.75 | 14.46 |
| 4w | 81.99 | 2.62 | 79.80 | 6.83 | 60.86 | 2.82 | 63.42 | 7.56 | 53.03 | 5.43 | 49.68 | 7.47 |
| 8w | 77.54 | 1.73 | 78.34 | 8.57 | 48.62 | 5.01 | 66.00* | 9.46 | 59.15 | 11.04 | 48.89 | 8.83 |
|  |  |  |  |  | *p<0.05, **p<0.01 vs same weeks ctrl. All data unit is degree. | | | | | | | |
