# Supplementary material for: Lower body kinematic changes induced by anterior cruciate ligament transection: an in vivo three-dimensional analysis in rats
Source: PeerJ. 2026 Mar 23;14:e21016. doi: 10.7717/peerj.21016 (PMC13020435; doi:10.7717/peerj.21016)
Supplement: Supplemental Information 3 [file peerj-14-21016-s003.docx]

Supplemental Table 2. Raw data of joint flexion range for 2D parameters in Figure 4.

|  | Ctrl | | | ACLT | | |
| --- | --- | --- | --- | --- | --- | --- |
| **Joint** | Q1 | Median | Q3 | Q1 | Median | Q3 |
| **Hip** |  |  |  |  |  |  |
| 1w | 16.51 | 19.36 | 21.90 | 24.95 | 27.16 | 31.02 |
| 2w | 20.42 | 23.18 | 25.23 | 18.62 | 24.70 | 29.47 |
| 4w | 19.73 | 20.84 | 22.12 | 17.43 | 20.49 | 22.92 |
| 8w | 21.17 | 22.53 | 23.86 | 17.19 | 19.34 | 21.77 |
| **Knee** |  |  |  |  |  |  |
| 1w | 68.12 | 73.60 | 80.48 | 54.00 | 55.89** | 59.60 |
| 2w | 69.84 | 72.22 | 75.83 | 64.59 | 67.80 | 70.72 |
| 4w | 65.82 | 67.95 | 68.56 | 58.86 | 62.68 | 68.15 |
| 8w | 63.61 | 66.22 | 73.74 | 61.71 | 64.71 | 71.56 |
| **Ankle** |  |  |  |  |  |  |
| 1w | 63.63 | 70.52 | 81.85 | 66.95 | 71.40 | 82.19 |
| 2w | 71.26 | 73.87 | 76.42 | 67.58 | 75.39 | 80.98 |
| 4w | 74.19 | 89.55 | 93.55 | 66.97 | 67.80 | 77.57 |
| 8w | 77.41 | 81.35 | 93.39 | 72.21 | 72.64 | 86.39 |
|  |  | Q1: The first quartile, Q3: the third quartile | | | | |
|  | **p<0.01 vs same weeks ctrl. All data unit is degree. | | | | | |
