# Supplementary material for: Lower body kinematic changes induced by anterior cruciate ligament transection: an in vivo three-dimensional analysis in rats
Source: PeerJ. 2026 Mar 23;14:e21016. doi: 10.7717/peerj.21016 (PMC13020435; doi:10.7717/peerj.21016)
Supplement: Supplemental Information 4 — Negative values represent numerical values in three-dimensional space and were switched to positive values for analysis. *p < 0.05 and **p < 0.01 indicate statistical significance compared with the control group in the same week for the range results. All data unit is degree. [file peerj-14-21016-s004.docx]

Supplemental Table 3. Raw data of 3D parameters in Figure 4.

|  | **Hip Abd/Add** | | | |  |  | **Knee inversion/valgus** | | | |  |  | **Pelvic tilt** | | | |
| --- | --- | --- | --- | --- | --- | --- | --- | --- | --- | --- | --- | --- | --- | --- | --- | --- |
|  | Ctrl | | ACLT | |  |  | Ctrl | | ACLT | |  |  | Ctrl | | ACLT | |
| **Abd** | mean | SD | mean | SD |  | **Inv** | mean | SD | mean | SD |  | **Right** | mean | SD | mean | SD |
| 1w | 35.89 | 5.46 | 42.45 | 12.35 |  | 1w | -71.03 | 21.42 | -24.77 | 15.85 |  | 1w | -16.10 | 11.00 | -12.5 | 5.11 |
| 2w | 36.42 | 4.61 | 36.27 | 7.36 |  | 2w | -75.63 | 30.60 | -46.58 | 21.79 |  | 2w | -15.81 | 6.16 | -15.05 | 5.84 |
| 4w | 35.56 | 3.85 | 37.01 | 8.02 |  | 4w | -57.28 | 6.85 | -55.03 | 24.15 |  | 4w | -20.03 | 6.22 | -15.85 | 8.69 |
| 8w | 30.31 | 3.76 | 33.70 | 5.98 |  | 8w | -81.17 | 18.19 | -62.57 | 25.82 |  | 8w | -17.83 | 1.73 | -21.46 | 6.45 |
| **Add** |  |  |  |  |  | **Val** |  |  |  |  |  | **Left** |  |  |  |  |
| 1w | -1.94 | 6.73 | 4.17 | 9.11 |  | 1w | 26.95 | 3.45 | 31.43 | 3.25 |  | 1w | 12.45 | 8.96 | 17.46 | 4.79 |
| 2w | -1.32 | 6.12 | 1.79 | 2.95 |  | 2w | 31.33 | 4.44 | 28.34 | 3.58 |  | 2w | 10.86 | 2.73 | 10.41 | 3.81 |
| 4w | 0.63 | 2.89 | -0.84 | 5.54 |  | 4w | 26.43 | 2.80 | 28.02 | 8.63 |  | 4w | 9.64 | 3.70 | 10.64 | 6.77 |
| 8w | -3.04 | 2.06 | -7.27 | 3.62 |  | 8w | 30.83 | 1.33 | 22.45 | 4.91 |  | 8w | 10.54 | 4.50 | 5.07 | 7.53 |
| **Range** |  |  |  |  |  | **Range** |  |  |  |  |  | **Range** |  |  |  |  |
| 1w | 37.83 | 2.30 | 38.28 | 9.68 |  | 1w | 97.98 | 24.49 | 56.21** | 14.55 |  | 1w | 28.55 | 2.15 | 29.96 | 3.32 |
| 2w | 37.74 | 8.73 | 34.48 | 5.58 |  | 2w | 106.96 | 29.01 | 73.32 | 22.95 |  | 2w | 26.67 | 3.67 | 25.45 | 4.13 |
| 4w | 34.94 | 4.69 | 37.85 | 6.02 |  | 4w | 83.72 | 4.57 | 83.04 | 20.61 |  | 4w | 29.67 | 2.78 | 26.48 | 5.10 |
| 8w | 33.34 | 2.07 | 40.97* | 4.75 |  | 8w | 112.00 | 18.23 | 84.21 | 29.80 |  | 8w | 28.37 | 2.77 | 26.52 | 4.32 |
|  |  |  |  |  |  |  | *p<0.05, **p<0.01: vs same weeks ctrl. All data unit is degree. | | | | | | | | | |
