# Supplementary figures and images for: Lower body kinematic changes induced by anterior cruciate ligament transection: an in vivo three-dimensional analysis in rats
Source: PeerJ. 2026 Mar 23;14:e21016. doi: 10.7717/peerj.21016 (PMC13020435; doi:10.7717/peerj.21016)

a

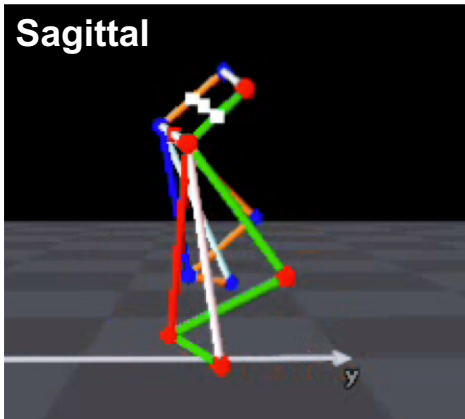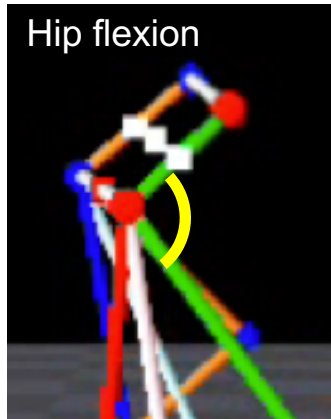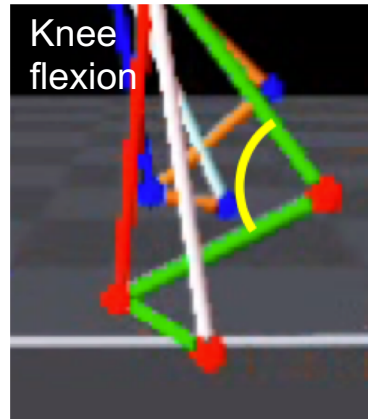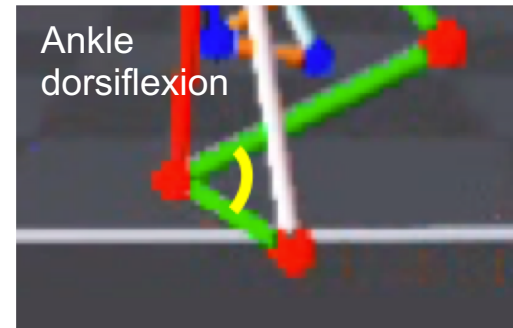

b

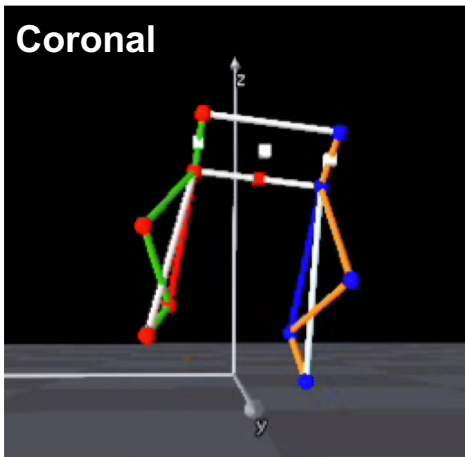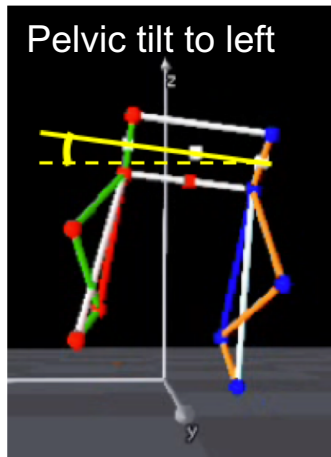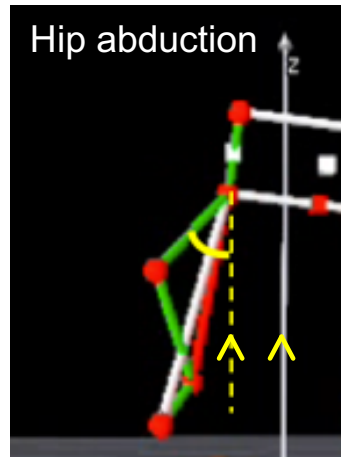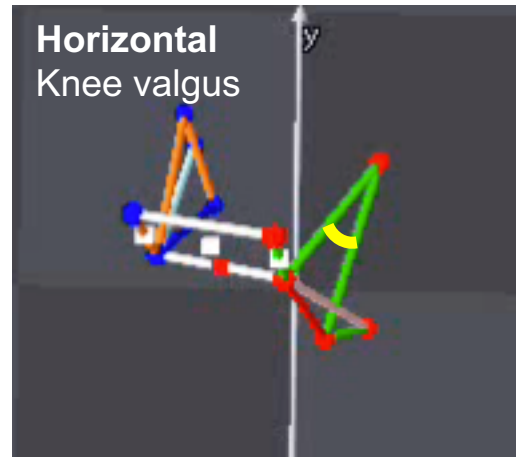

Supplement: Supplemental Information 5 — (a) 2D parameters on the sagittal plane; and (b) 3D parameters on the coronal and horizontal planes. The yellow circular line indicates the measured angle. On the sagittal plane, three parameters: hip flexion, knee flexion, and ankle angle, were analyzed. On the coronal plane, two parameters: pelvic tilt to the right/left and hip abduction/adduction were analyzed. On the horizontal plane, knee inversion/valgus were analyzed. [file peerj-14-21016-s005.pdf]

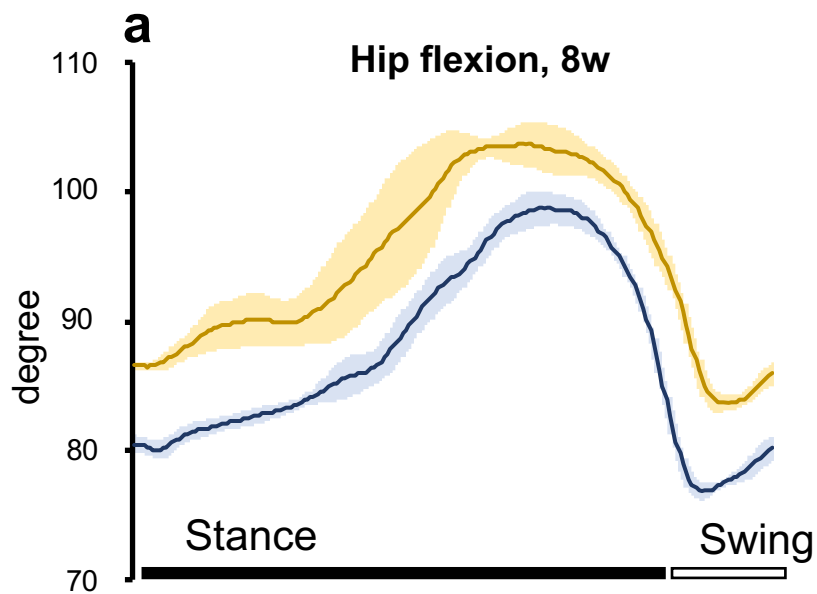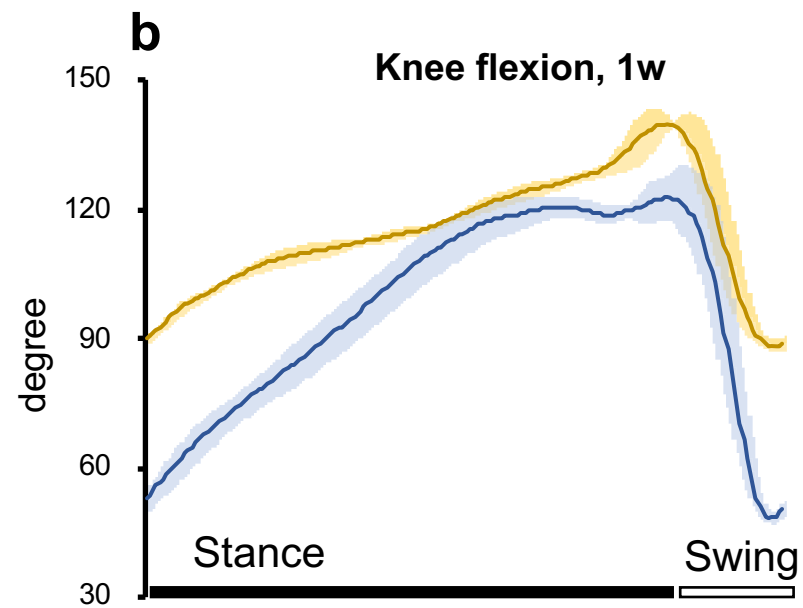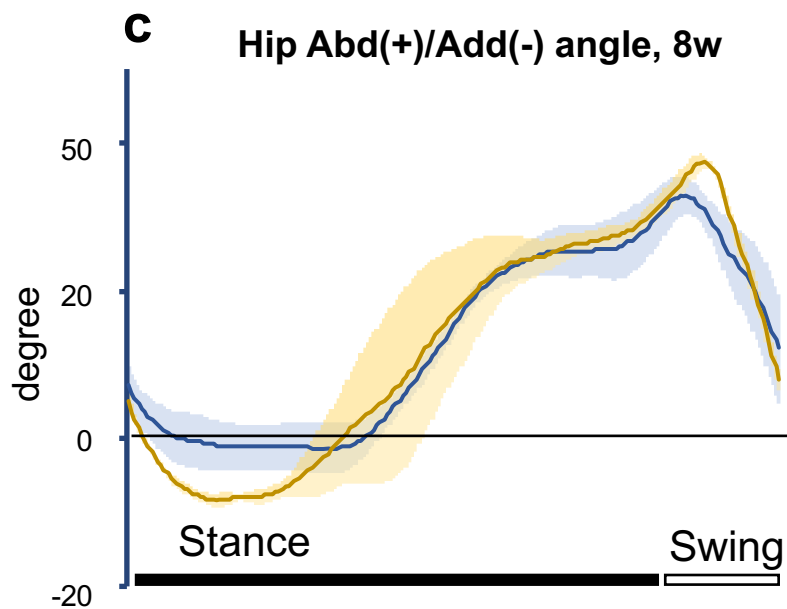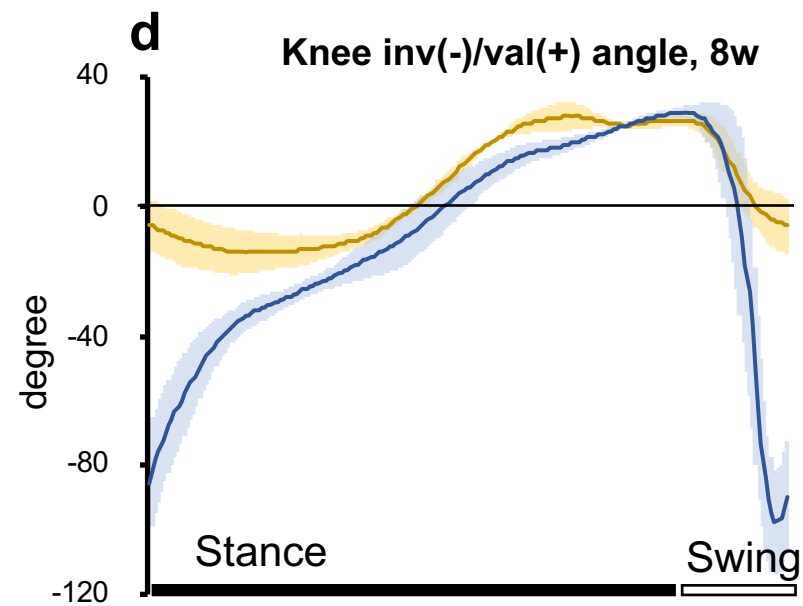

— Ctrl — ACLT

Supplement: Supplemental Information 6 — (a) The representative trajectory of hip flexion range at eight weeks showing a similar trajectory and lower angle range in the ACLT group compared to the Ctrl. (b) The representative trajectory of knee flexion range at one week showing a higher angle and lower angle range in the ACLT group compared to the Ctrl. (c) The trajectory of hip abduction/adduction at eight weeks showing the peak of the abduction angle (maximum) in the swing phase and peak of adduction angle (minimum) in the stance phase of ACLT are larger than that of the Ctrl. (d) The trajectory of knee valgus/inversion range at eight weeks showing lower inversion and valgus angles and a lower angle range in the ACLT group compared to the Ctrl. [file peerj-14-21016-s006.pdf]
